# Supplementary material for: Androgen receptor and MYC equilibration centralizes on developmental super-enhancer
Source: Nat Commun. 2021 Dec 15;12:7308. doi: 10.1038/s41467-021-27077-y (PMC8674345; doi:10.1038/s41467-021-27077-y)
Supplement: Supplementary file 1 — Supplementary Information [file 41467_2021_27077_MOESM1_ESM.pdf]

SUPPLEMENTARY TABLE 1. PRIMERS AND PROBES SEQUENCES

(1) 3C droplet digital PCR (ddPCR) primers and probes:
FAM-MYC-Pst-QSY FAM-TGCAGCCTGGTACGCGCGTGGCGT-QSY (Probe)
q3C-MYC-pro-PstI-C GTATAAATCATCGCAGGCGGAAC (constant primer)
Copy number reference: HEX-RPP30-Iowa Black (from Bio-Rad Catalog number 10033173)

Locus-specific primer (span from the left to the right arms of 8q24 locus)

q3C-Site-1	TGAATCACTGACAGAGCCCAGTT
q3C-Site-2	CTGATCCACCGCTGCTCTCTGAA (PCAT1#1)
q3C-Site-3	TGGGGAACCCAAATAACAGACCA (PCAT1#2)
q3C-Site-4	ACTCTGCAACCATCTGCATTCCTA
q3C-Site-5	GAAATGACAATAGAAATCTTTGTTAC (PCAT1#3)
q3C-Site-6	CAAGGTGCCGGTACTAAGAAGTGG
q3C-Site-7	AAATAAATAACCTGTAAAATCAC
q3C-Site-8	AAAAAGGTGTGGGAGAAGAAGGAA
q3C-Site-9	CAACCGCAAGGTACACAGGAAAT (ABS#1)
q3C-Site-10	CTCAATCCACAGTGACAGCAGGA (ABS#2)
q3C-Site-11	TGTTCTCCAAGAAAGTTCAGTAAGGT (ABS#3)
q3C-Site-12	GCACACCTGTGAAGCTGTTGTGT
q3C-Site-13	CCTTGCCAATGCTGAATATGGTT

(2) 4C-Seq PCR amplification primers:
Round-1 PCR primers:
The forward primer: 4C-MYC-Pro-Dpn-F CCCGGGTTCCCAAAGCAGAGG
The reverse primer: 4C-MYC-Pro-Pst-R CGCACTGCGCGCCCACCGC

Round-2 PCR primers:
The forward primer: 4C-Read1-Adaptor-F5:
ACACTCTTTCCCTACACGACGCTCTTCCGATCTAGAGGGCGTGGGGGAAAAGAAAAA
The reverse primer: 4C-Read2-Adaptor-R5:
GACTGGAGTTCAGACGTGTGCTCTTCCGATCTCACGCCACGCGCGTACCAGGC

(3) ChIP-qPCR primers:

PSA-Enh-F	TGGGACAACTTGCAAACCTG
PSA-Enh-R	CCAGAGTAGGTCTGTTTTCAATCCA
TMPRSS2-Enh-F	GTGCACTGTCCTCTGCCTTGCT
TMPRSS2-Enh-R	CACCAGGTGGCCATTTGTTGTC

(4) RT-qPCR primers:

MYC-5UTR-F	AAGGGAGATCCGGAGCGAATAG
MYC-5UTR-R	CTGCTATGGGCAAAGTTTCGTG
MYC-E2-E3-F	CAGCGACTCTGAGGAGGAACAA
MYC-E2-E3-R	CTCCAGCAGAAGGTGATCCAGA
GAPDH-F	TTGCCATCAATGACCCCTTCA
GAPDH-R	CGCCCCACTTGATTTTGGA

Supplementary Table S2 Data sets used in this study.

Cell/tissue type and treatment	Data type	Data Availability
VCaP (10 nM DHT, 0, 4, 10, 24 hr)	RNA-Seq	GEO: GSE157107 (This work)
VCaP siCtrl and siMYC (Androgen starvation)	RNA-Seq	GEO: GSE157107 (This work)
VCaP siCtrl and siMYC (FBS)	RNA-Seq	GEO: GSE157107 (This work)
LNCaP-AR (10 nM DHT, 0, 24 hr)	RNA-Seq	GEO: GSE157107 (This work)
LNCaP-AR and LNCaP-AR-MYC (FBS)	RNA-Seq	GEO: GSE157107 (This work)
VCAP (10 nM DHT 0, 4 hr)	MYC ChIP-Seq	GEO: GSE157107 (This work)
VCAP (10 nM DHT 0, 2, 24 hr)	H3K27ac ChIP-Seq	GEO: GSE157107 (This work)
VCAP (FBS, FBS+DHT, FBS+DHT+ENZ)	H3K27ac ChIP-Seq	GEO: GSE157107 (This work)
VCaP (10 nM DHT, 0, 2 hr)	4C-Seq	GEO: GSE157107 (This work)
VCaP (10 nM DHT, 0, 2, 24 hr)	H3K27ac HiChIP (Chr8)	GEO: GSE157107 (This work)
LNCaP (1 nM DHT, 0, 24 hr)	Affymetrix U133A microarray (Transcriptome)	PMID: 16885382
LNCaP-AR (1 nM R1881, 0, 24 hr)	Illumina HumanHT-12 V3.0 (Transcriptome)	GSE62474
VCaP (1 nM DHT, 0, 24 hr)	Illumina HumanHT-12 V4.0 (Transcriptome)	GSE62473
LNCaP (10 nM DHT, 0, 24 hr)	RNA-Seq	GSE125014
LNCaP (10 nM DHT, 0, 24 hr)	RNA-Seq	GSE114267
LNCaP (10 nM DHT, 0, 24 hr)	RNA-Seq	PMID: 28916652
LNCaP-AR (2 nM R1881, 0, 24 hr)	RNA-Seq	GSE120660
VCAP (10 nM DHT, 0, 12 hr)	AR ChIP-Seq	GSE55062
VCAP (10 nM DHT, 0, 12 hr)	BRD4 ChIP-Seq	GSE55062
LNCAP (100nM, 0, 2 hr)	AR ChIP-Seq	GSE83860
Benign prostate tissues	RNA-Seq	TCGA-PRAD
Primary PCa tissues	RNA-Seq	TCGA-PRAD
CRPC tissues	RNA-Seq	dbGAP: phs001648.v1.p1
Benign prostate tissues	H3K27ac ChIP-Seq	GSE130408
Primary PCa tissues	H3K27ac ChIP-Seq	GSE130408
CRPC PDXs	H3K27ac ChIP-Seq	GSE130408
Benign prostate tissues	AR ChIP-Seq	GSE130408
Primary PCa tissues	AR ChIP-Seq	GSE130408
CRPC PDXs	AR ChIP-Seq	GSE130408
Benign prostate tissues	FOXA1 ChIP-Seq	GSE130408
Primary PCa tissues	FOXA1 ChIP-Seq	GSE130408
CRPC PDXs	FOXA1 ChIP-Seq	GSE130408
Benign prostate tissues	HOXB13 ChIP-Seq	GSE130408
Primary PCa tissues	HOXB13 ChIP-Seq	GSE130408
CRPC PDXs	HOXB13 ChIP-Seq	GSE130408
VCAP (10 nM R1881 0, 2 hr)	CTCF ChIP-Seq	GSE84432
VCaP xenograft (Pre-castration and One day after castration)	RNA-Seq	GSE56829
LNCaP clone FGC	Hi-C	GSE105557
VCaP siCtrl and siAR (10 nM DHT, 0, 24 hr)	RNA-Seq	GSE82223
pan-Cancer tissues	ATAC-Seq	TCGA
pan-Cancer cell lines	H3K27ac ChIP-Seq	ENCODE
VCaP (FBS)	H3K27ac ChIP-Seq	GSE96652
LNCaP (10nM DHT, 0, 4hr)	H3K27ac ChIP-Seq	GSE51621
208 pairs of primary PCa and adjacent tissues	Whole-genome bisulfite sequencing	GSA: PRJCA001124
5 prostate cancers, 4 matched benign prostate tissues adjacent to tumor,	Whole-genome bisulfite sequencing	PMID: 24113458
VCAP (10nM R1881, 30min, 2hr)	GRO-Seq	GSE84432

Supplementary Figure 1

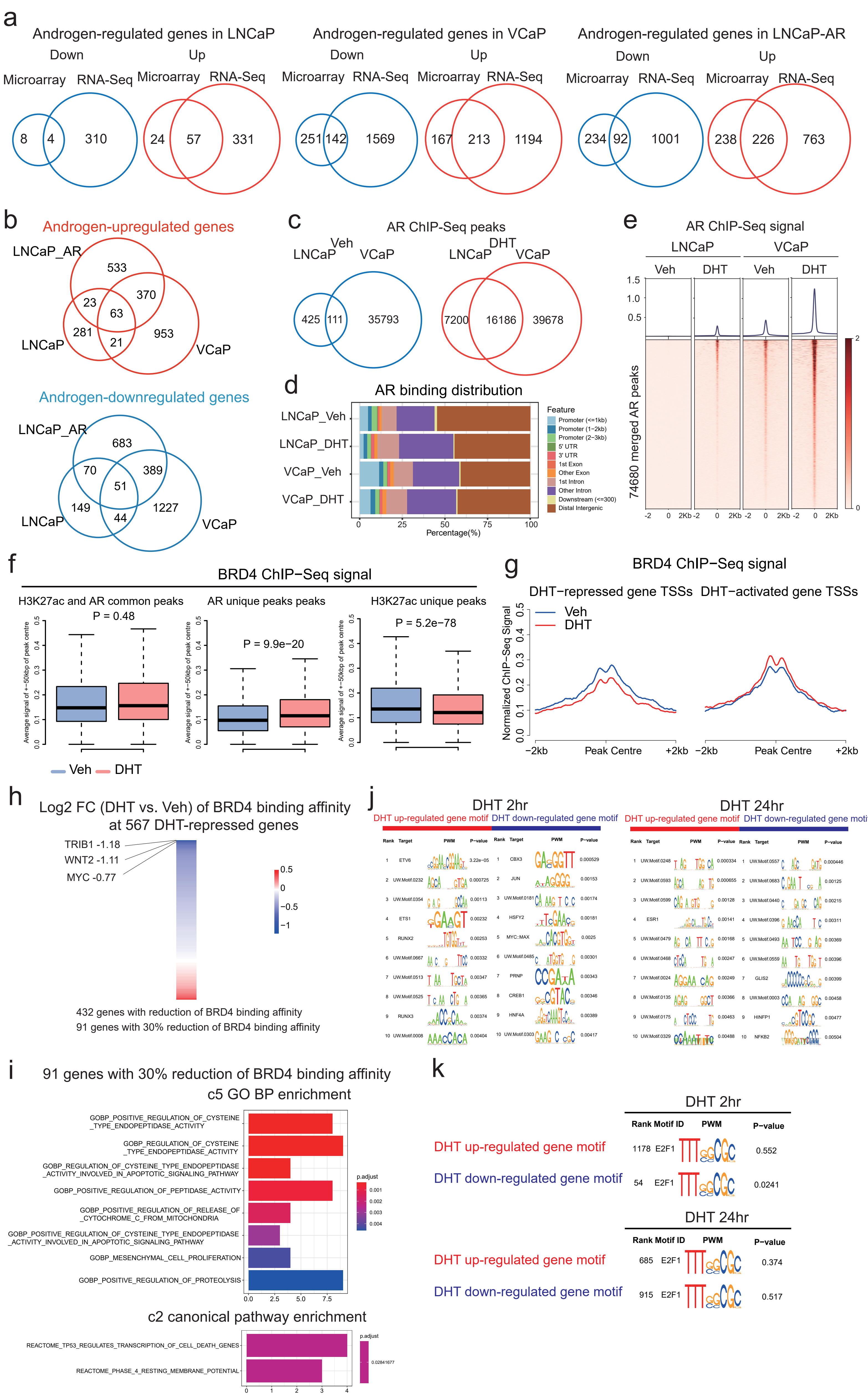

Supplementary Figure S1. Androgen stimulation effects on chromatin binding and gene expression.

(a) Venn diagrams showing the overlap of DHT-regulated genes between microarray data sets and RNA-seq data sets in Figure 1C.

(b) Venn diagrams showing overlap of DHT-upregulated and DHT-downregulated genes in each cell line from RNA-seq data.

(c) Venn diagrams showing the overlapping of AR peaks between VCaP (GSE55062) and LNCaP (GSE83860) cells under vehicle or DHT condition.

(d) Genomic distribution of AR peaks in VCaP and LNCaP cells.

(e) Pileup plots and heatmaps showing AR binding intensity in VCaP and LNCaP cells under vehicle or DHT condition.

(f) Boxplot showing the BRD4 ChIP-seq signal at the centers of H3K27ac/AR common peaks, AR-specific peaks and H3K27ac-specific peaks. The average signal of 100 bp region surrounding peak center was summoned. Box limits, 1st and 3rd quartiles; whiskers, 1.5x inter-quartile range; center line, median. From the left to right, n = 11344, 11344, 37313, 37313, 75966 and 75966, respectively. P-values were determined by two-sided Student's t-test.

(g) Average ChIP-seq signal of BRD4 around TSSs of DHT-repressed genes and DHT-activated genes. ± 2kb regions of peak centers were binned by 50 bp windows.

(h) Log2 fold changes (DHT vs. Veh) of BRD4 binding intensity at DHT-repressed gene TSSs were ordered from lowest to highest. Top 3 genes were highlighted together with their BRD4 binding log2 fold changes.

(i) The genes with DHT-induced expression downregulation and > 30% BRD4 binding reduction were subjected to functional enrichment analysis in MSigDB gene set c2 and c5.

(j) Motif enrichment analysis of H3K27ac peaks annotated to DHT-upregulated genes or DHT-downregulated genes at early (2 hr) and late (24 hr) time points in VCaP. H3K27ac peaks within ± 20kb region of gene TSS were selected. R package "PWMEnrich" was used to conduct motif enrichment analysis.

(k) An enrichment of E2F1 motif that is known to mark the promoter of cell cycle genes was only identified at the DHT-repressed signatures genes at 2 hours, as shown by the p-value.

Supplementary Figure 2

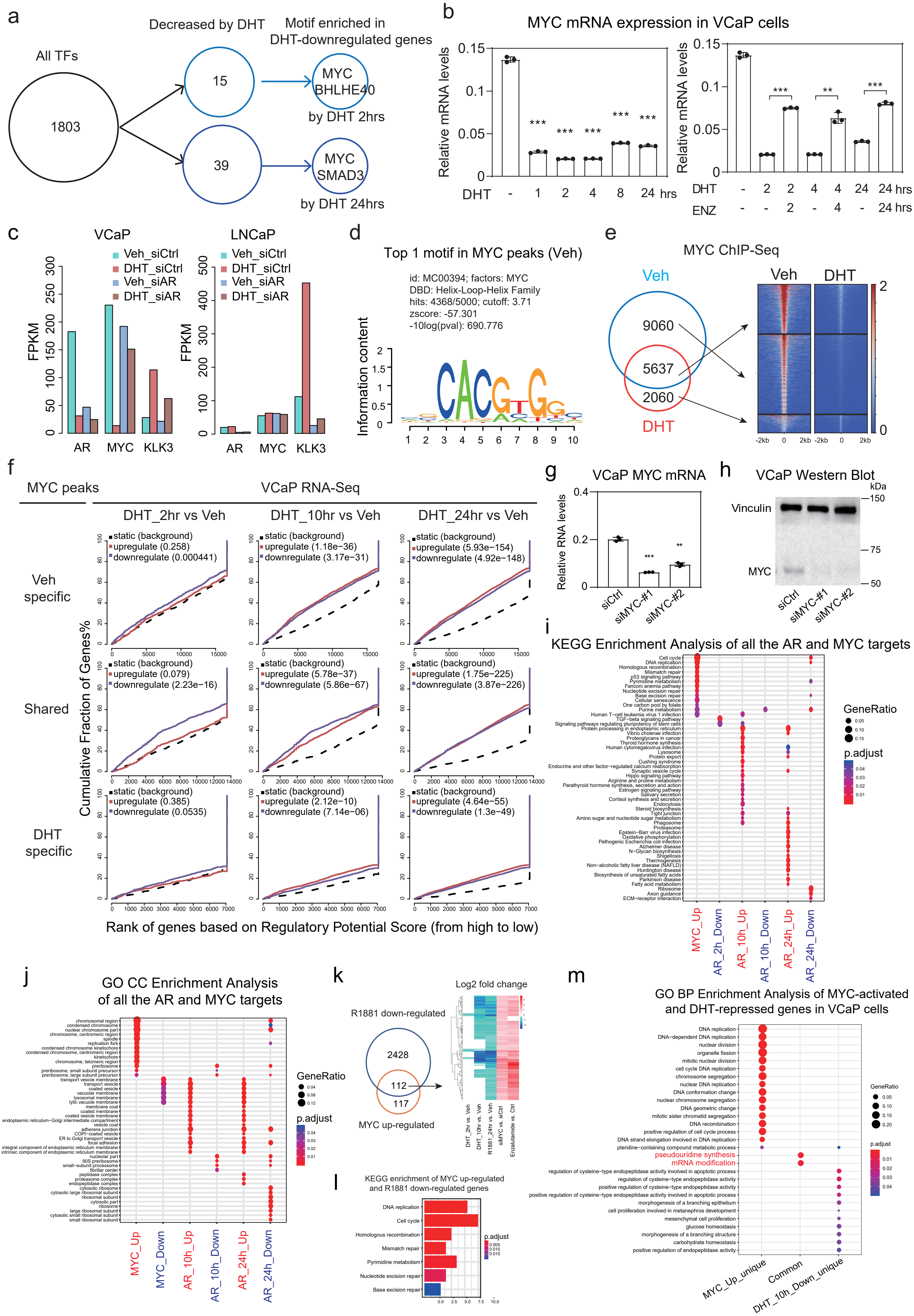

Supplementary Figure S2. Androgen effects on MYC expression and regulated genes.
(a) Expression changes of TF genes (n=1803) in VCaP cells that were repressed by DHT (fold change < 2/3 and P < 0.05) at 2 and 24 hrs.
(b) VCaP cells in androgen-depleted medium were treated with DHT +/- ENZ, and MYC was assessed by qRT-PCR (normalized to GAPDH internal control).
(c) Effects of AR knockdown by siAR and DHT on AR, MYC, and KLK3/PSA expression in VCaP and LNCaP cells (GSE82223). The siAR was from Thermofisher/Ambion (Catalog, s1538) that targets AR exon-2. It was shown to specifically attenuate AR mRNA in RT-PCR analyses [1] and AR proteins in Western blotting [2].
(d) Top 1 enriched motif from MYC ChIP-seq peaks in VCaP cells under vehicle condition.
(e) Venn diagram and heatmap showing the overlap between MYC binding peaks under vehicle condition and after DHT-stimulation in VCaP cells.
(f) BETA to determine the association between MYC binding (vehicle-specific, DHT-specific, and common peaks) and DHT-driven gene expression at different time points in VCaP cells. Red line, DHT-upregulated genes; Purple line, DHT-downregulated genes.
(g-h) qRT-PCR and Western blotting of VCaP cells to assess the knockdown efficacy of two independent siRNAs targeting MYC. RNA from these samples was subsequently examined by RNA-seq.
(i-j) KEGG pathway and GO enrichment analysis of genes regulated by MYC and AR.
(k) Significant overlap between MYC-upregulated and R1881 down-regulated genes (2 nM R1881 for 24hrs, GSE120660).
(l) KEGG enrichment of MYC upregulated and R1881 down-regulated genes (2 nM R1881 for 24hrs, GSE120660).
(m) GO enrichment analysis of genes upregulated by MYC or/and downregulated by DHT in Figure 2m. The enriched GO terms of overlapping genes were highlighted in red color.
For (b) and (g), data are presented as mean values +/- SD of 3 biological (b) or technical (g) replicates. P-values were determined by two-sided Student's t-test. N.S., not significant; *, P < 0.05; **, P < 0.01; ***, P < 0.001.
Source data are provided in Immunoblot and Histogram Source Data files.

Supplementary Figure 3

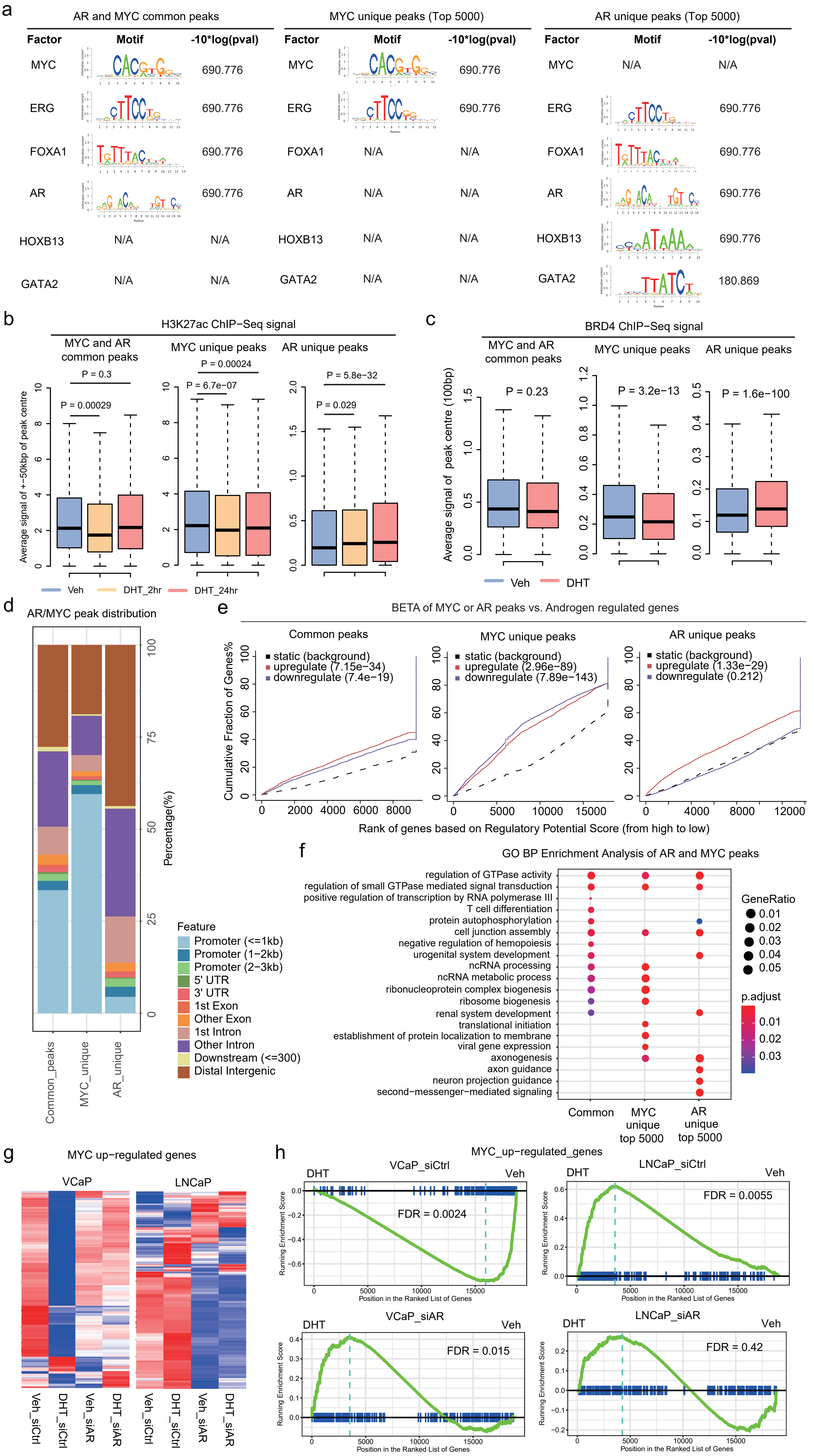

Supplementary Figure S3. Characterization of MYC and AR common versus unique binding sites.

(a) Motif enrichment analysis of AR-specific, MYC-specific, and AR-MYC common peaks in VCaP cells. Only the ERG/ETS motif was enriched in all 3 subgroups. Notably, the FOXA1 motif, but not the HOXB13 or GATA2 motifs, were enriched in the AR unique and AR/MYC common sites.

(b-c) Boxplots showing the H3K27ac or BRD4 ChIP-seq signal at the centers of MYC/AR common peaks, MYC-specific peaks and AR-specific peaks. The average signal of 100 bp region surrounding peak center was summoned. Box limits, 1st and 3rd quartiles; whiskers, 1.5x inter-quartile range; center line, median. From the left to right, n = 3716, 3716, 3716, 10981, 10981, 10981, 52149, 52149, 52149, 3716, 3716, 10981, 10981, 52149 and 52149, respectively. P-values were determined by two-sided Student's t-test.

(d) Genomic distribution of AR/MYC common peaks, MYC unique peaks and AR unique peaks. As noted, MYC is characterized as a TSS-proximal transcriptional regulator, AR is primarily a distal regulator, while their common sites are both proximal and distal.

(e) BETA to determine the association between AR/MYC binding (common, MYC unique and AR unique) and DHT-responsive gene expression in VCaP cells. The red line indicates DHT-upregulated genes and the purple line indicates the DHT-downregulated genes.

(f) GO BP enrichment analysis of AR/MYC common peaks, MYC unique peaks and AR unique peaks. Genes associated with ChIP-seq peaks were used to calculate the GO term enrichment score. It should be noted ribosome biogenesis programs are enriched in both MYC-specific and common signatures.

(g) Heatmap analyses of the effects of AR knockdown by siAR and of DHT on MYC-activated genes in VCaP and LNCaP cells. The LNCaP and VCaP siAR data are based on datasets GSE82223.

(h) GSEA analyses of the effects of AR knockdown by siAR and of DHT on MYC-activated genes in VCaP and LNCaP cells. The LNCaP and VCaP siAR data are based on datasets GSE82223.

Supplementary Figure 4

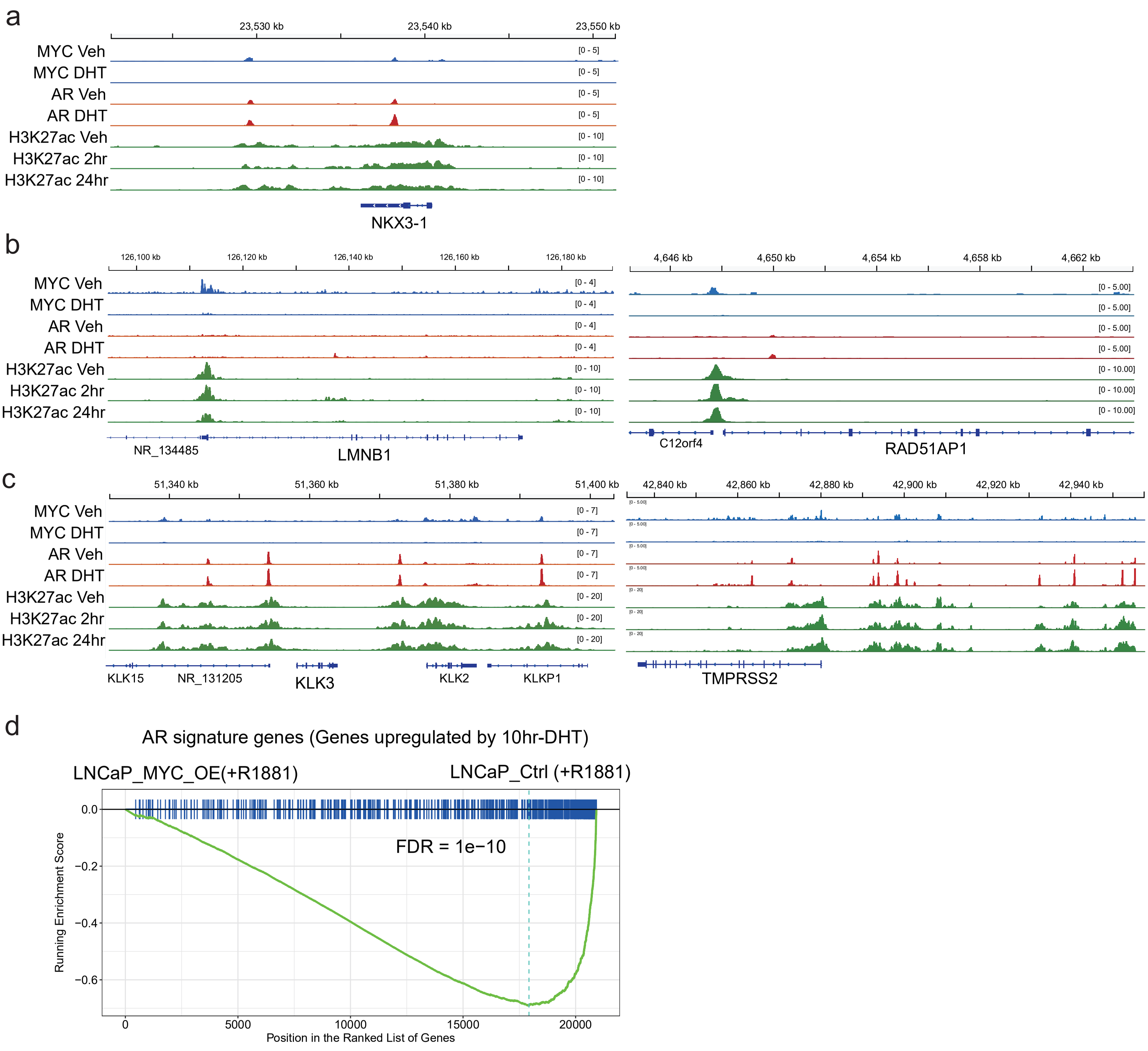

Supplementary Figure S4. MYC, AR, and H3K27Ac at selected AR and MYC regulated genes.

(a-c) AR/MYC binding alignments at an AR/MYC common direct target gene (*NKX3-1*), two MYC unique direct target genes (*LMNB1* and *RAD51AP1*), and two AR unique target genes (*KLK3* and *TMPRSS2*).

(d) GSEA of the gene sets upregulated by 10 hr DHT treatment (10 nM) in LNCaP cells to assess the effects of doxycycline-induced MYC overexpression (MYC_OE) on AR transactivation (GSE73917).

Supplementary Figure 5

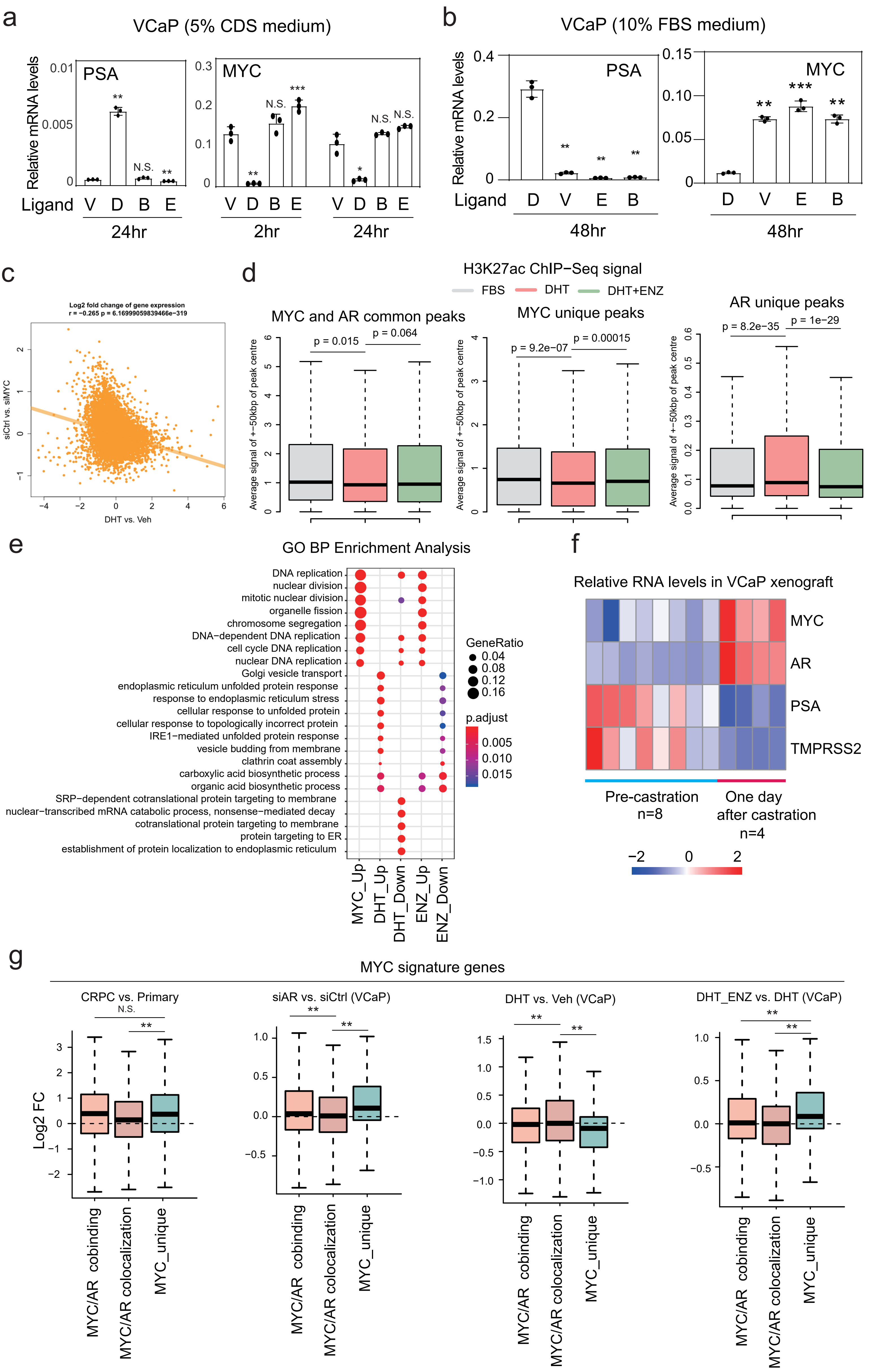

Supplementary Figure S5. Associations between MYC and AR binding sites and transcriptional activity.

(a) VCaP cells in medium with steroid depleted FBS (5% CDS medium) were treated with vehicle, DHT (D, 10 nM), bicalutamide (B, 10 μM), or enzalutamide (10 μM) for 2 or 24 hours. Expression of KLK3/PSA and MYC mRNA was then assessed by qRT-PCR. Data are presented as mean values +/- SD for 3 biologically independent samples.
(b) VCaP cells in medium 10% FBS were treated with vehicle, DHT (D, 10 nM), bicalutamide (B, 10 μM), or enzalutamide (10 μM) for 48 hours. Expression of KLK3/PSA and MYC mRNA was then assessed by qRT-PCR. Data are presented as mean values +/- SD for 3 biologically independent samples.
(c) Scatter plot showing a global inverse correlation between MYC-regulated expression changes and DHT-elicited expression changes in VCaP.
(d) Boxplot showing the H3K27ac ChIP-Seq signal at the centers of AR/MYC-common peaks, AR-specific peaks and MYC-specific peaks. The average signal of 100 bp region surrounding peak center was summoned. Box limits, 1st and 3rd quartiles; whiskers, 1.5x inter-quartile range; center line, median. From the left to right, n = 3716, 3716, 3716, 10981, 10981, 10981, 52149, 52149 and 52149, respectively. P-values were determined by two-sided Student's t-test.
(e) Robust common GO terms enrichment in MYC-upregulated genes and ENZ-upregulated genes.
(f) Normalized messages of MYC, AR, and classic AR targets (PSA and TMPRSS2) in VCaP xenografts at one day after castration (GSE56829).
(g) Log2 fold changes of MYC target genes in different comparisons. MYC target genes were separated into three subgroups based on MYC and AR binding status at ± 20 kb region of gene TSS. Box limits, 1st and 3rd quartiles; whiskers, 1.5x inter-quartile range; center line, median. MYC/AR cobinding, n = 47; MYC/AR colocalization, n = 76; MYC unique, n = 47.
For (a), (b) and (g), P-values were determined by two-sided Student's t-test. N.S., not significant; *, $P < 0.05$; **, $P < 0.01$; ***, $P < 0.001$.
Source data are provided in Histogram Source Data file.

Supplementary Figure 6

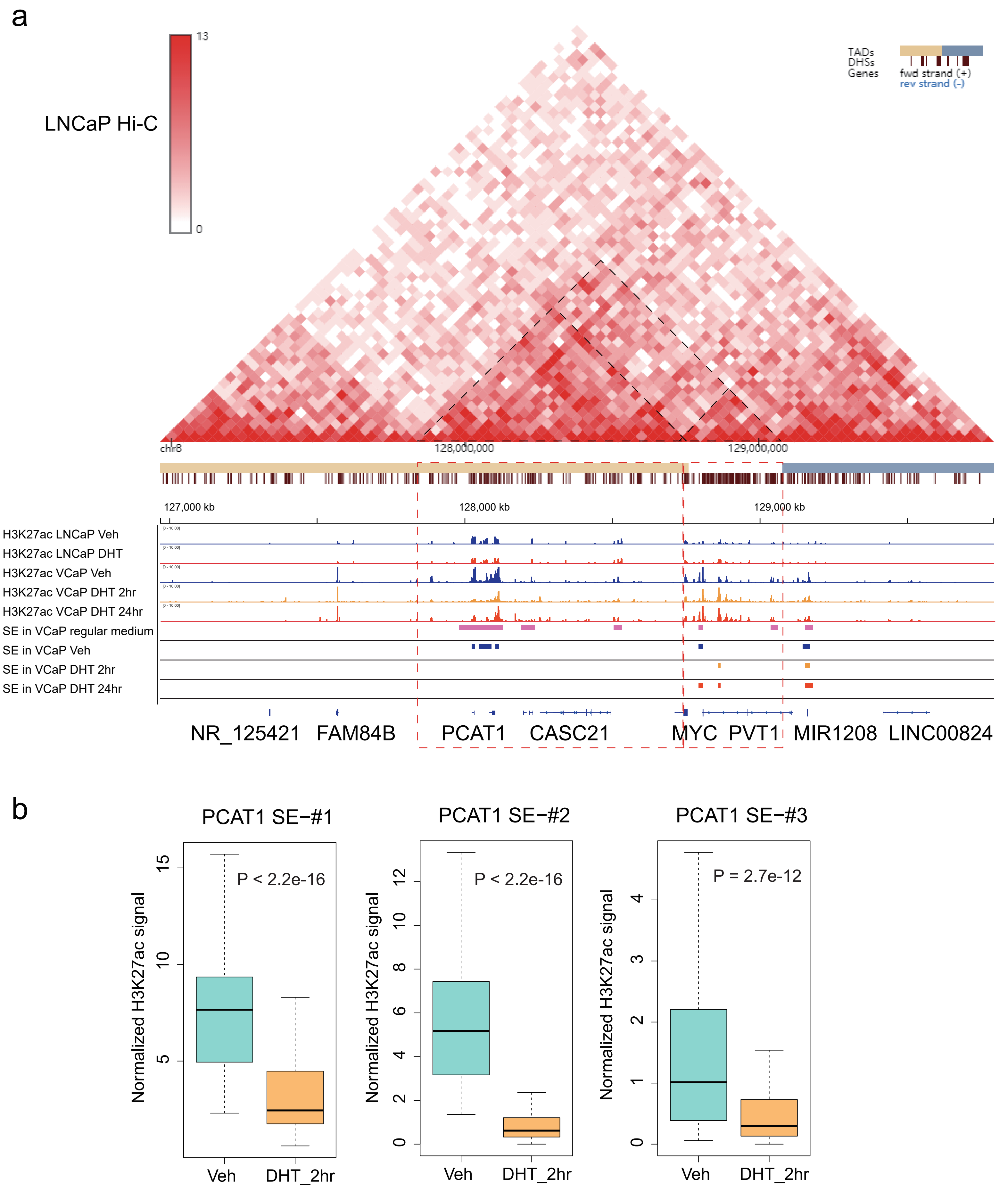

Supplementary Figure S6. Structural organization of 8q24 locus.

(a) LNCaP Hi-C data (GSE105557) showing *MYC* is in the same TAD with *PCAT1* SEs. The bottom is an alignment of LNCaP (GSE51621) and VCaP enhancer (as marked by H3K27ac) distribution with and without androgen. As framed, the 8q24 locus is architecturally organized as a TAD that actually contains two sub-TADs, on the left and right arms of *MYC*, respectively. Enhancers on the left arm were substantially repressed by androgen in VCaP, while those on the right arm were less inhibited. In LNCaP cells with lower AR expression, enhancers in this locus were not markedly perturbed. These findings are consistent with androgen effects on expression of MYC and its flanking lncRNAs in these two cell lines.

(b) Each of the three PCAT1 sub-SE (as highlighted above in S6A) was divided into 100 bins, and the average H3K27ac ChIP-Seq signal of each bin was extracted from bigWig files for box plots and two-sided paired t-tests. Box limits, 1st and 3rd quartiles; whiskers, 1.5x inter-quartile range; center line, median. For each box, n =100.

Supplementary Figure 7

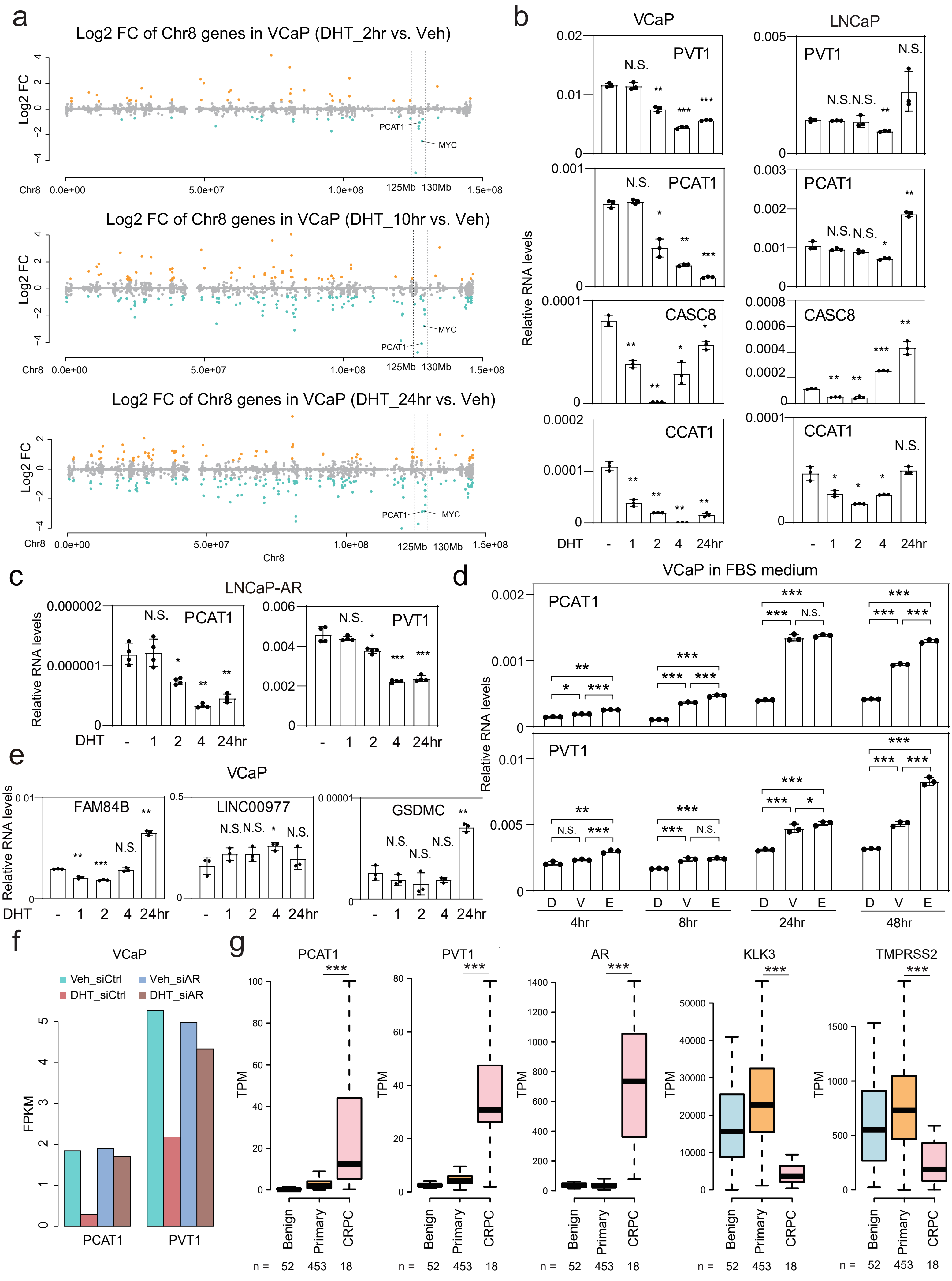

Supplementary Figure S7. Androgen suppresses transcription across 8q24 locus.
(a) Log2 fold changes of transcripts (including both coding genes and lncRNAs) upon DHT stimulation of VCaP at different time points across chromosome 8. The region of high interaction with PCAT1 SEs was highlighted by dotted lines.
(b) VCaP and LNCaP cell lines were subjected to 10 nM DHT treatment for indicated time points. Total RNA was submitted to TaqMan RT-PCR analyses of indicated MYC-flanking lncRNAs that are located in the 8q24 TAD.
(c) LNCaP-AR cell lines were subjected to 10 nM DHT treatment for indicated time points. Total RNA was submitted to TaqMan RT-PCR analyses of PCAT1 and PVT1 expression.
(d) VCaP cells were adapted in androgen-proficient medium (10% FBS + 10 nM DHT, D) and then switched to lower androgen levels (10% FBS + vehicle, V) or anti-androgen treatment (10% FBS + 10 μM of ENZ, E) for indicated time points. Total RNA was subjected to RT-qPCR analysis of PCAT1 and PVT1 expression.
(e) VCaP cells were subjected to 10 nM DHT treatment for indicated time points. Total RNA was subjected to TaqMan RT-PCR analyses of indicated genes that locate at the left (*FAM84B*) and right (*LINC00977* and *GSDMC*) boundaries of the 8q24 TAD, respectively.
(f) Effects of AR knock-down by siAR and of androgen on PCAT1 and PVT1 expression in VCaP cells (GSE82223).
(g) Indicated gene expression in benign prostate tissues, primary PCa and CRPC. In the primary PCa and CRPC cases, tumors with AR and/or MYC amplification were excluded. Box limits, 1st and 3rd quartiles; whiskers, 1.5x inter-quartile range; center line, median. Benign prostate tissues, n = 52; primary PCa, n= 453; CRPC, n = 18.
For (b-e), data are presented as mean values +/- SD; for each test, n=3 biologically independent samples. For (b), (c), (e) and (g), P-values were determined by two-sided Student's t-test. N.S., not significant; *, $P < 0.05$; **, $P < 0.01$; ***, $P < 0.001$.
Source data are provided in Histogram Source Data file.

Supplementary Figure 8

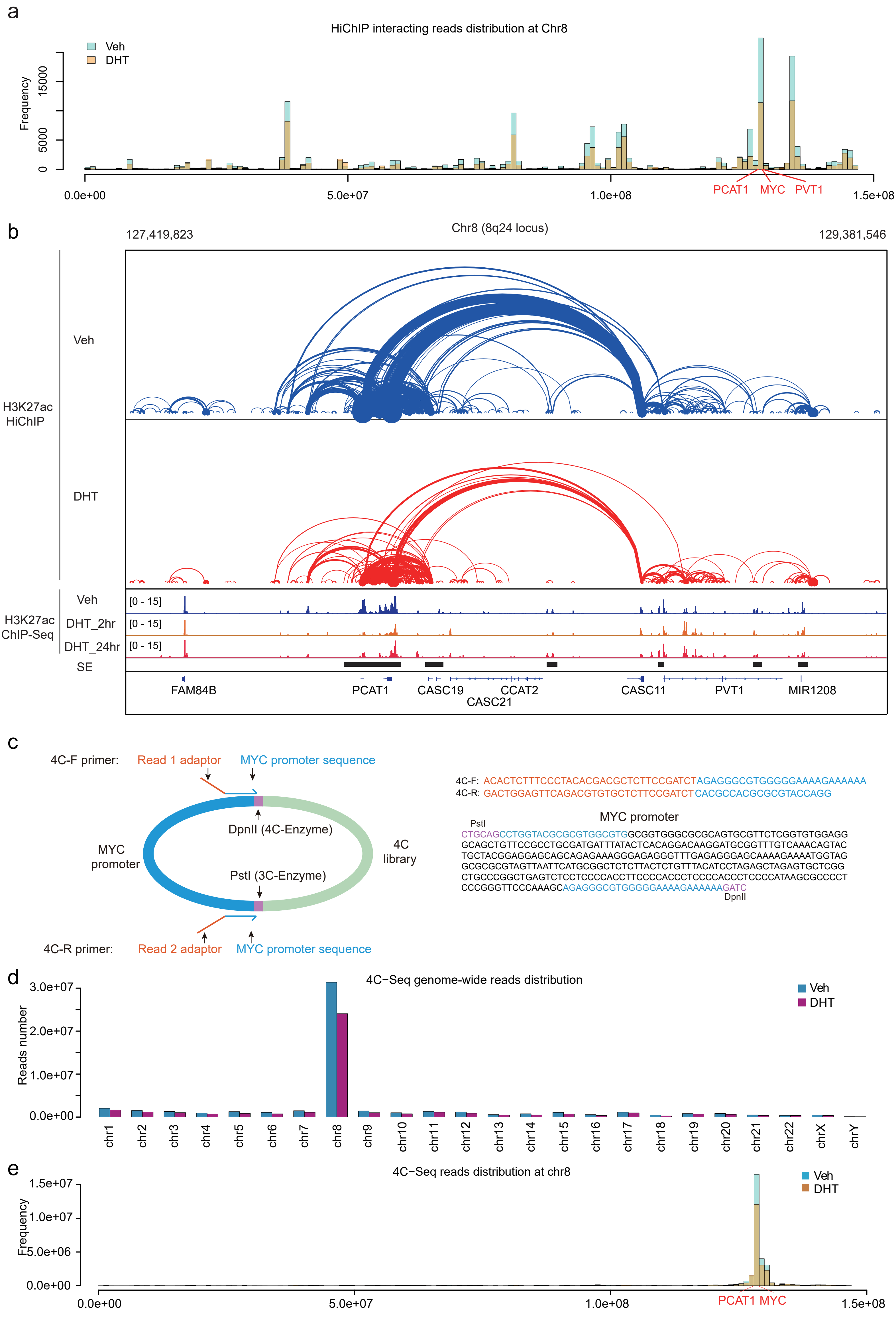

Supplementary Figure S8. MYC promoter interactions with sites across 8q24 locus.

(a) VCaP H3K27ac HiChIP mated read distribution at chromosome 8. Light blue bar indicates the frequency of mated reads under vehicle condition and orange bar indicates the frequency of mated reads under DHT condition. The 8q24 locus, as the top interacting region, was highlighted.

(b) All the significant H3K27ac HiChIP loops in the *PCAT1*/*MYC* region in VCaP are shown. The loop strengths are normalized by total valid pair-end read number. This display allows better appreciation of the androgen effects on the looping across the 8q24 locus and its immediate genomic vicinity.

(c) Experimental design outlining 4C-Seq library construction to screen *MYC* promoter interactome.

(d) Genome-wide *MYC* promoter-anchored 4C-Seq reads distribution across all human chromosomes in VCaP cells, which indicated an exclusive interaction of *MYC*-promoter within chromosome 8 (chr8).

(e) Similar as (d), 4C-Seq read distribution on chr8 that indicates *MYC*-promoter interactions are robustly enriched in the 8q24 locus and repression by DHT of its interaction with the *PCAT1* region.

Supplementary Figure 9

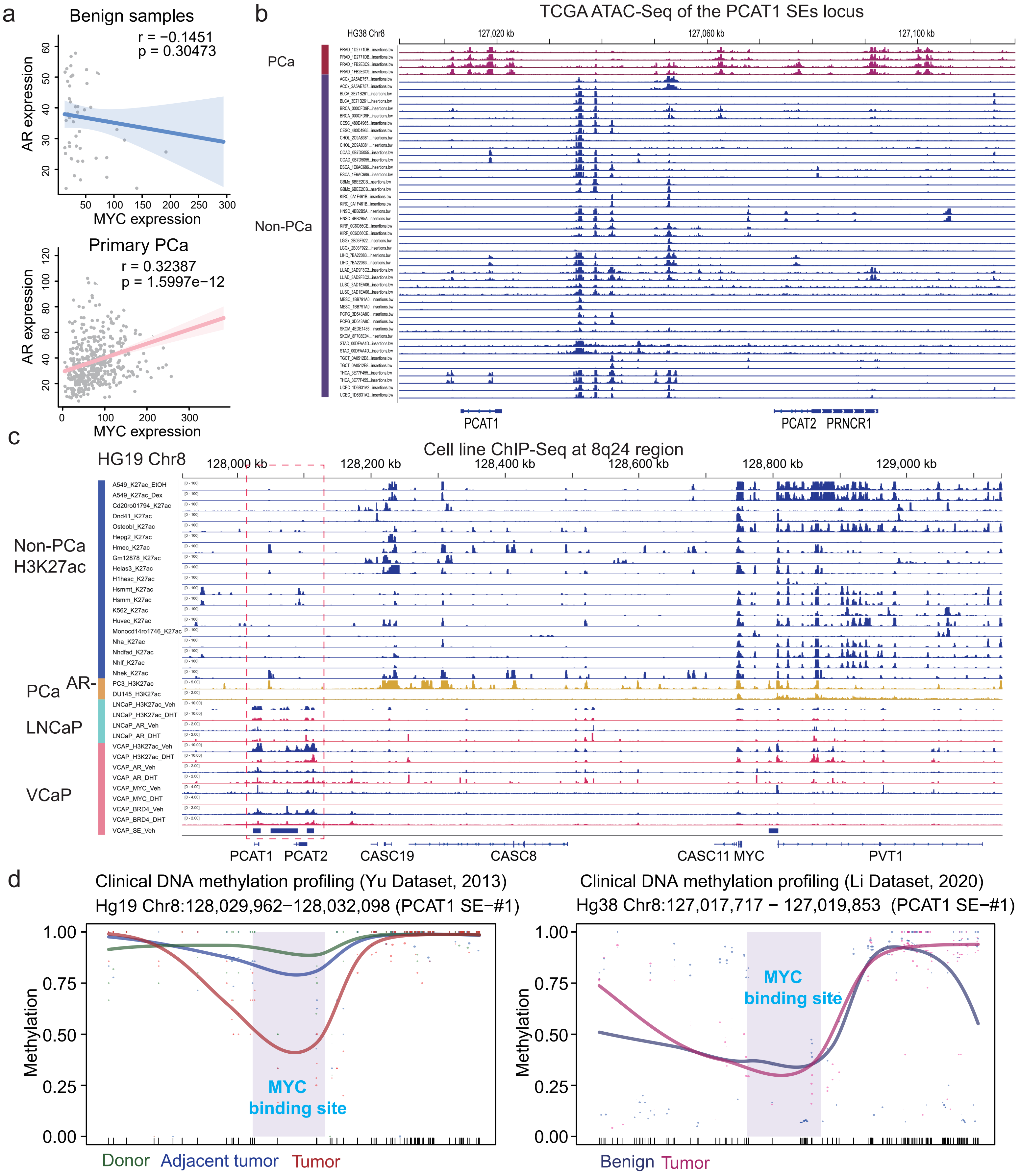

Supplementary Figure S9. Epigenetic features of 8q24 locus in diverse cell types and clinical samples.

(a) Pearson's correlation between AR and MYC expression based on benign prostate tissues and primary PCa. In the primary PCa, AR and/or MYC amplification cases were excluded.

(b) ATAC-seq tracks from TCGA tumor samples showing chromatin accessibility at PCAT1 SE region. Four samples were randomly chosen for PCa ATAC-Seq data and a panel of additional samples were randomly chosen for each of other tumor types.

(c) ChIP-seq tracks from ENCODE cell lines showing H3K27ac signal at 8q24 locus. The *PCAT1* SE, located at ~700 Kb upstream of the MYC promoter, was highlighted by a dashed frame. As shown, the *PCAT1* region has high levels of H3K27ac only in the AR expressing PCa cells with corresponding AR binding, suggesting a stimulatory and developmental role of AR in activation of this enhancer. In VCaP cells, the *PCAT1* SE is also occupied by MYC. Notably, these SEs are decreased by DHT in the AR-high VCaP cells but not in LNCaP cells with lower AR expression.

(d) Annotation of VCaP MYC ChIP-Seq and clinical DNA methylation profiling of the *PCAT1*-SE based on an American cohort (left panel) and an Asia cohort (right panel). Highlighted is the region of MYC occupancy.

Supplementary Figure 10

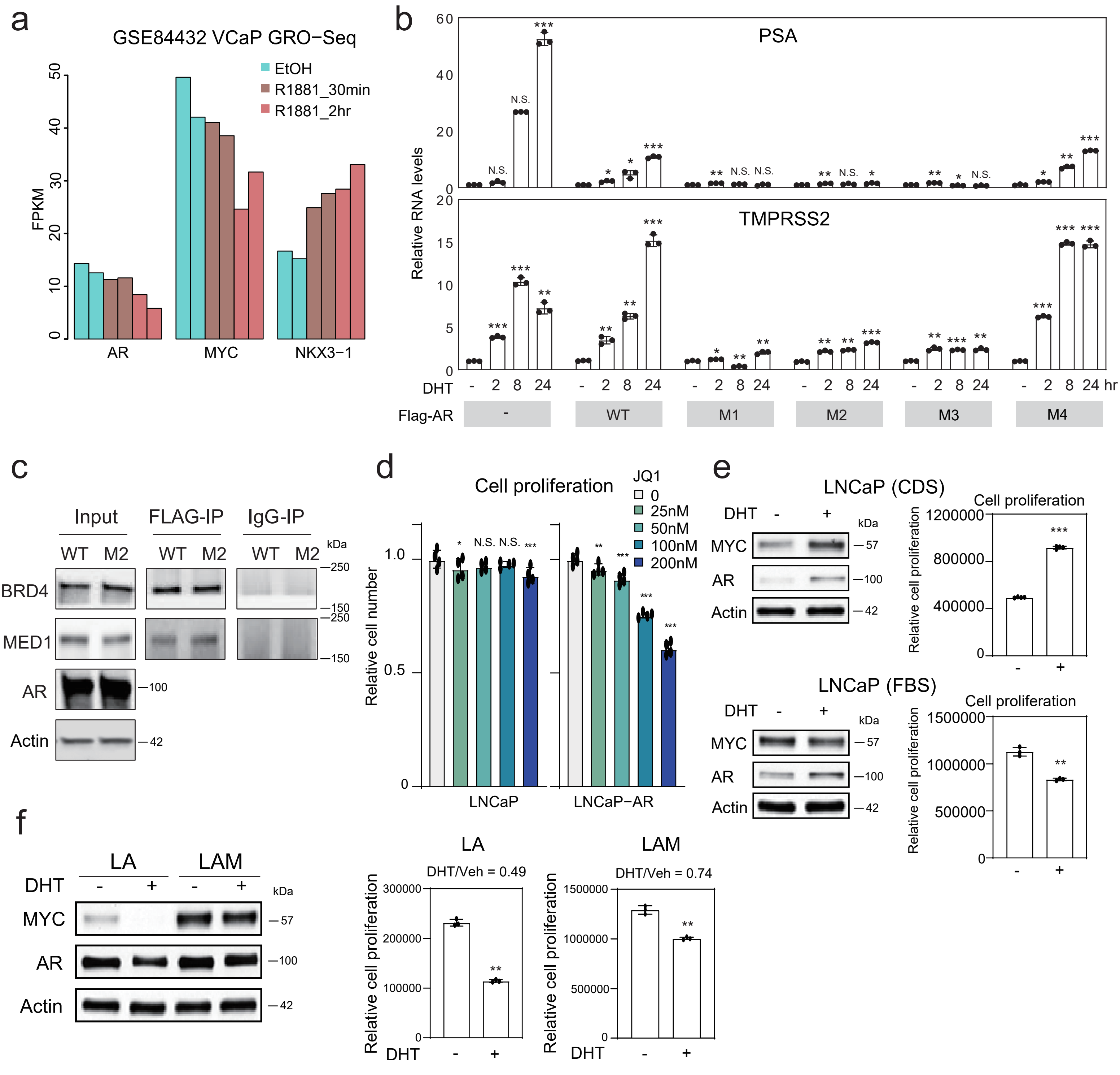

Supplementary Figure S10. Activities of wildtype versus DNA binding mutant AR.
(a) Gro-Seq analysis of VCap cells treated with androgen (R1881) (GSE84432).
(b) LNCaP cell stably overexpressing expressing WT or mutant ARs were stimulated with DHT as in Figure 7G, and analyzed by qRT-PCR for PSA/KLK3 and TMPRSS2 mRNA.
(c) The LNCaP-AR stable lines overexpressing Flag-tagged AR wild-type (WT) versus the DBD mutant (M2, C619Y) in complete medium were lysed and immunoprecipitated with anti-Flag antibody versus control IgG. The input, Flag-IP samples, and control IgG samples were then Western blotted for BRD4 and MED1.
(d) LNCaP and LNCaP-AR were cultured in FBS medium and treated with low range of JQ1 doses for 3 days. Cell proliferation was assessed using the CellTiterGlo kit.
(e) LNCaP cells were cultured in androgen-depleted (CDS) or androgen-containing (FBS) medium and treated without or with DHT (10 nM). Upon 24 hrs proteins were harvested for Western blotting and upon 3 days cell proliferation assay was carried out using the CellTiterGlo kit.
(f) LNCaP-AR (LA, for AR overexpression) and LNCaP-AR-MYC (LAM for AR and MYC overexpression) were cultured in FBS medium and treated with or without DHT (10 nM). Upon 24 hrs, proteins were harvested for Western blotting (left); upon 3 days cell proliferation assay was carried out using the CellTiterGlo kit (right).
The Western blots in (c), (e), and (f) are representative of 3 biological replicates. For (b), (d), (e), and (f), data are presented as mean values +/- SD of 3-4 biological replicates. P-values were determined by two-sided Student's t-test. N.S., not significant; *, $P < 0.05$; **, $P < 0.01$; ***, $P < 0.001$.
Source data are provided in Histogram and Immunoblot Source Data files.

Supplementary Figure 11

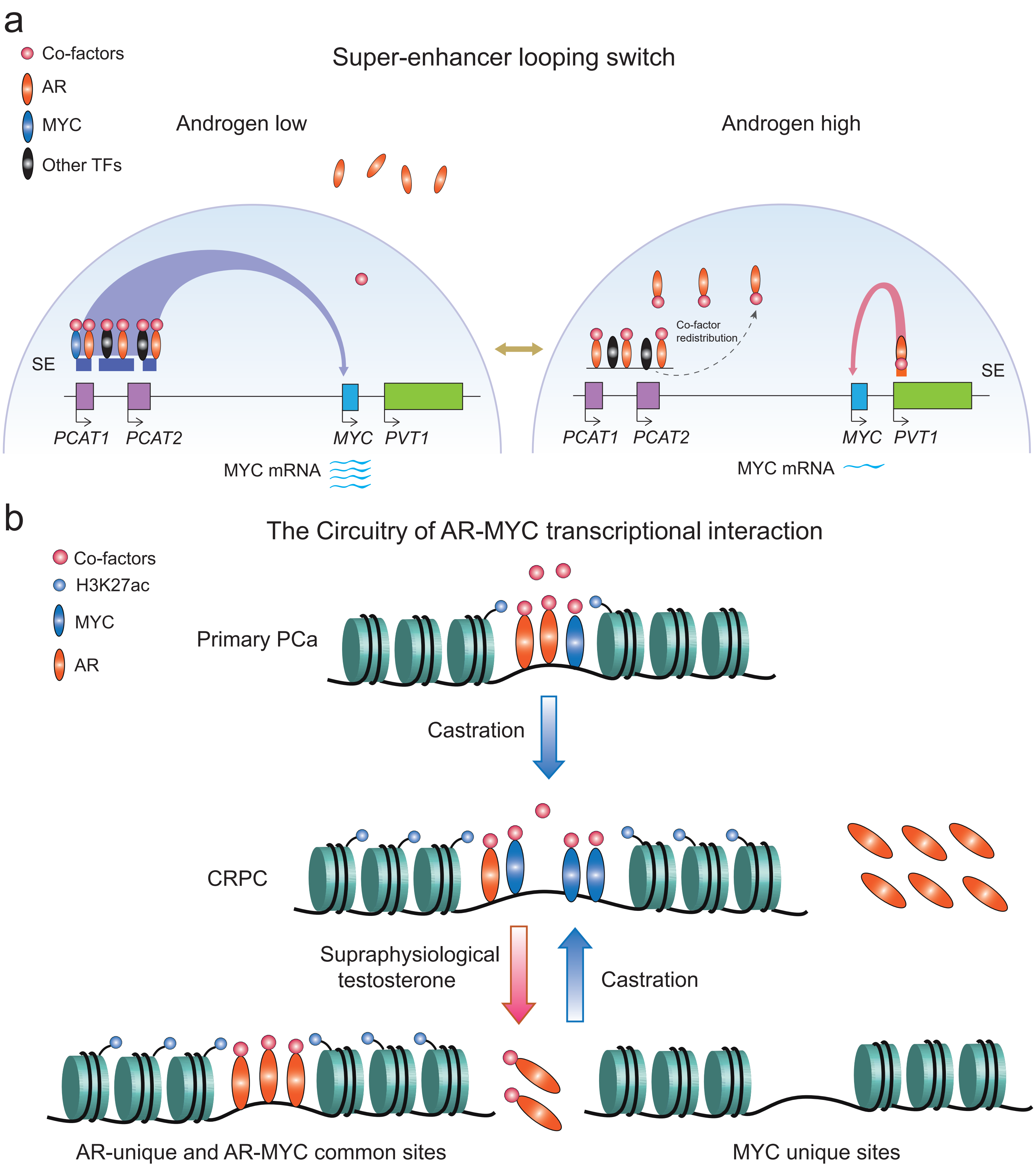

Supplementary Figure S11. Graphical abstract of AR – MYC interactions in response to androgen stimulation or deprivation.

Model a: Architectural regulation of MYC by distal enhancers in the 8q24 locus. In prostate the entire 8q24 locus is architecturally organized as a TAD, with the *MYC* promoter looping to enhancers clustered in the left arm (*PCAT1* region) and right arm (*PVT1* region). The *MYC* super-enhancer overlapping the *PCAT1* and *PCAT2* genes (PCAT1 SEs) is activated specifically during PCa development in association with AR occupancy. Transcriptionally, the *MYC* gene is constantly equilibrated by the dual functions of AR on the *PCAT1* SE: a direct positive regulation and an indirect negative regulation. While the direct function requires intact AR, the indirect effect depends on AR nuclear localization but not DNA binding. Specifically, in AR-high cells, coactivator redistribution upon androgen stimulation inactivates the *PCAT1*-SE due to loss of coactivators from other TFs. This is partially compensated by a shift in the docking of the *MYC* promoter to the enhancers in the right arm (*PVT1* SEs), which is less active and leads to locus-wide repression. Mechanistically, the convergence of the dual AR regulatory signals in this locus can buffer and equilibrate the expression of MYC, which has a short half-life at both message and protein levels.

Model b: Circuitry of AR-MYC transcriptional interaction. A subset of AR regulated genes in primary PCa are coregulated by MYC. AR activation of these genes is diminished after castration, but this is compensated by an increase in MYC. Moreover, the increase in MYC also increases expression of MYC unique genes (not shown), which together compromise the efficacy of castration therapy. In CRPC cells expressing high levels of AR, treatment with supraphysiological testosterone can restore or enhance expression of genes regulated by AR alone or by AR and MYC, but acutely decrease genes regulated by MYC alone. This acute loss of MYC may then contribute to the efficacy of supraphysiological testosterone in men with CRPC.

Supplementary References

1. Misawa, A., Takayama, K., Urano, T. & Inoue, S. Androgen-induced Long Noncoding RNA (lncRNA) SOCS2-AS1 Promotes Cell Growth and Inhibits Apoptosis in Prostate Cancer Cells. *J Biol Chem* **291**, 17861-17880 (2016).
2. Takayama, K.I. *et al.* Integrative Genomic Analysis of OCT1 Reveals Coordinated Regulation of Androgen Receptor in Advanced Prostate Cancer. *Endocrinology* **160**, 463-472 (2019).
