# Supplementary material for: Androgen receptor and MYC equilibration centralizes on developmental super-enhancer
Source: Nat Commun. 2021 Dec 15;12:7308. doi: 10.1038/s41467-021-27077-y (PMC8674345; doi:10.1038/s41467-021-27077-y)
Supplement: Supplementary file 3 — Reporting Summary [file 41467_2021_27077_MOESM3_ESM.pdf]

nature portfolio

Corresponding author(s): Housheng Hansen He, Shaoyong Chen, Steven P. Balk

Last updated by author(s): Nov 3, 2021

Reporting Summary

Nature Portfolio wishes to improve the reproducibility of the work that we publish. This form provides structure for consistency and transparency in reporting. For further information on Nature Portfolio policies, see our Editorial Policies and the Editorial Policy Checklist.

Statistics

For all statistical analyses, confirm that the following items are present in the figure legend, table legend, main text, or Methods section.

n/a	Confirmed	
☐	☒	The exact sample size (*n*) for each experimental group/condition, given as a discrete number and unit of measurement
☐	☒	A statement on whether measurements were taken from distinct samples or whether the same sample was measured repeatedly
☐	☒	The statistical test(s) used AND whether they are one- or two-sided<br>*Only common tests should be described solely by name; describe more complex techniques in the Methods section.*
☐	☒	A description of all covariates tested
☐	☐	A description of any assumptions or corrections, such as tests of normality and adjustment for multiple comparisons
☐	☒	A full description of the statistical parameters including central tendency (e.g. means) or other basic estimates (e.g. regression coefficient) AND variation (e.g. standard deviation) or associated estimates of uncertainty (e.g. confidence intervals)
☐	☒	For null hypothesis testing, the test statistic (e.g. *F*, *t*, *r*) with confidence intervals, effect sizes, degrees of freedom and *P* value noted<br>*Give P values as exact values whenever suitable.*
☒	☐	For Bayesian analysis, information on the choice of priors and Markov chain Monte Carlo settings
☒	☐	For hierarchical and complex designs, identification of the appropriate level for tests and full reporting of outcomes
☒	☐	Estimates of effect sizes (e.g. Cohen's *d*, Pearson's *r*), indicating how they were calculated

Our web collection on statistics for biologists contains articles on many of the points above.

Software and code

Policy information about availability of computer code

Data collection	No software was used to collect data in this study.
Data analysis	Data analysis were conducted mostly by R language. STAR, BOWTIE2 and MACS2 were used for next generation sequencing data analysis (See Methods section).<br>The custom scripts used in this study is available at https://github.com/Haiyangg/Script_for_MYC_project<br>Raw sequencing reads were first trimmed by Trim Galore (https://github.com/FelixKrueger/TrimGalore)

For manuscripts utilizing custom algorithms or software that are central to the research but not yet described in published literature, software must be made available to editors and reviewers. We strongly encourage code deposition in a community repository (e.g. GitHub). See the Nature Portfolio guidelines for submitting code & software for further information.

Data

Policy information about availability of data

All manuscripts must include a data availability statement. This statement should provide the following information, where applicable:
- Accession codes, unique identifiers, or web links for publicly available datasets
- A description of any restrictions on data availability
- For clinical datasets or third party data, please ensure that the statement adheres to our policy

The high throughput sequencing data reported in this paper have been deposited to GEO under accession number GSE157107.
The additional data sets used below are all publicly available.
AR, FOXA, HOXB13 and H3K27ac ChIP-Seq data of PCa tissuses are from GSE130408 (https://www.ncbi.nlm.nih.gov/geo/query/acc.cgi?acc=GSE130408).
TCGA-PRAD RNA-Seq data from Genomic Data Commons Data Portal (https://portal.gdc.cancer.gov/).

Quigley CRPC RNA-seq data is available at http://davidquigley.com/prostate.html.
RNA-Seq of VCaP cells with AR knockdown is from GSE82223 (https://www.ncbi.nlm.nih.gov/geo/query/acc.cgi?acc=GSE82223).
VCaP xenograft RNA-Seq data is from GSE56829 (https://www.ncbi.nlm.nih.gov/geo/query/acc.cgi?acc=GSE56829).
LNCaP RNA-Seq data is from GSE125014 (https://www.ncbi.nlm.nih.gov/geo/query/acc.cgi?acc=GSE125014) and GSE114267(https://www.ncbi.nlm.nih.gov/geo/query/acc.cgi?acc=GSE114267).
LNCaP Hi-C data is from GSE105557 (https://www.ncbi.nlm.nih.gov/geo/query/acc.cgi?acc=GSE105557).
ATAC-Seq data of TCGA pan-cancer tissues are available at https://gdc.cancer.gov/about-data/publications/ATACseq-AWG.
H3K27ac ChIP-Seq in cancer cell lines are downloaded from ENCODE (https://www.encodeproject.org/chip-seq-matrix/?type=Experiment&replicates.library.biosample.donor.organism.scientific_name=Homo%20sapiens&assay_title=Histone%20ChIP-seq&assay_title=Mint-ChIP-seq&status=released).
Whole-genome bisulfite sequencing data of PCa tissues are from https://ngdc.cncb.ac.cn/gsa-human/browse/HRA000099.
VCaP GRO-Seq data is from https://www.ncbi.nlm.nih.gov/geo/query/acc.cgi?acc=GSE84432.
AR and BRD4 ChIP-Seq data of VCaP cells are from GSE55062 (https://www.ncbi.nlm.nih.gov/geo/query/acc.cgi?acc=GSE55062).
AR ChIP-Seq data of LNCaP cells is from GSE83860 (https://www.ncbi.nlm.nih.gov/geo/query/acc.cgi?acc=GSE83860)

Field-specific reporting

Please select the one below that is the best fit for your research. If you are not sure, read the appropriate sections before making your selection.

[x] Life sciences [ ] Behavioural & social sciences [ ] Ecological, evolutionary & environmental sciences

For a reference copy of the document with all sections, see nature.com/documents/nr-reporting-summary-flat.pdf

Life sciences study design

All studies must disclose on these points even when the disclosure is negative.

Sample size	In order to determine differences in MYC expression noted and based on the variability between individual mice we estimated the number of animals per experimental group for xenograft studies needed to provide statistical significance based on a student "t" test.
Data exclusions	No data was excluded from the analyses.
Replication	For each experiment, all attempts at replication were successful.
Randomization	Randomization was not considered within the experimental groups tested.
Blinding	The animal experiments were conducted as a blinded study by technicians and were unknowing of the origins of the cells being tested.

Reporting for specific materials, systems and methods

We require information from authors about some types of materials, experimental systems and methods used in many studies. Here, indicate whether each material, system or method listed is relevant to your study. If you are not sure if a list item applies to your research, read the appropriate section before selecting a response.

Materials & experimental systems

n/a	Involved in the study	
[ ]	[x]	Antibodies
[ ]	[x]	Eukaryotic cell lines
[x]	[ ]	Palaeontology and archaeology
[ ]	[x]	Animals and other organisms
[x]	[ ]	Human research participants
[x]	[ ]	Clinical data
[x]	[ ]	Dual use research of concern

Methods

n/a	Involved in the study	
[ ]	[x]	ChIP-seq
[x]	[ ]	Flow cytometry
[x]	[ ]	MRI-based neuroimaging

Antibodies

Antibodies used

MYC Santa Cruz sc-764 (ChIP-Seq, 4ug; IHC, 1:2,500)
MYC Abcam ab32072 (ChIP-qPCR, 4ug; Western blotting, 1:1,000)
AR Santa Cruz sc-816 (HiChIP, 5ug)
AR Abcam Ab74272 (ChIP-qPCR, 4ug; Western blotting, 1:1,000)
H3K27Ac Abcam ab4729 (ChIP-Seq, 4ug; HiChIP, 5ug)
H3K27Ac DIAGENODE Cat. C15410196 (ChIP-qPCR, 4ug)
Beta-Actin (Abcam, Cat. Ab6276, Western blotting, 1:1,000)

Flag-M2 (Sigma Aldrich, Cat. F3165, Western blotting, 1:1,000)
MED1 (Bethyl, A300-793A, Western blotting, 1:1,000)
Control IgGs (Santa Cruz: normal rabbit IgG, Cat. sc-2027, ChIP-qPCR, 4ug)

Validation

The antibodies have been validated in other studies, the link of which could be found in the manufacturer's website.

Eukaryotic cell lines

Policy information about cell lines

Cell line source(s)

LNCaP ATCC CRL-1740
VCaP ATCC CRL-2876

Authentication

We utilized ATCC services following extended passages to authenticate by utilizing Short Tanden Repeat (STR) profiling. Sequences were amplified 17 STR loci plus Amelogenin using Promega's PowerPlex® 18D System. A comprehensive analysis report interprets both karyotypically normal and abnormal cell lines, includes a electropherograms supporting the allele calls at each locus, known reference profiling against the ATCC STR database and a comprehensive interpretation of results.

Mycoplasma contamination

All cell lines were tested for mycoplasma contamination using MycoAlert™ Mycoplasma Detection Kit (LT07-118, Lonza). No mycoplasma contamination was detected in these cell lines.

Commonly misidentified lines
(See ICLAC register)

No cell lines used in this study are listed in the database of commonly misidentified cell lines maintained by ICLAC.

Animals and other organisms

Policy information about studies involving animals; ARRIVE guidelines recommended for reporting animal research

Laboratory animals

Six- to eight-week-old male ICR/scid mice (IcrTac:ICR-Prkdc<scid>) from Taconic (Taconic Biosciences, Inc., 273 Hover Avenue, Germantown, NY 12526, USA) were used to generate PCa xenografts. The mice are housed in Allentown HEPA-filtered, individually ventilated cages with ducted HEPA exhaust and automatic watering. Irradiated food and hyperchlorinated, reverse osmosis water are provided to all cages. Rodent racks, cages, and bedding are autoclaved in double-door bulk autoclaves prior to use. The mice were injected subcutaneously with 2 million VCaP cells in 50% Matrigel. When Xenografts reached ~1000 mm3, biopsies were obtained. Additional biopsies were obtained 4 d after castration, and the tumors were harvested at relapse (3 weeks).

Wild animals

The study did not involve any wild animal.

Field-collected samples

The study did not involve any sample collected from the field.

Ethics oversight

All animal experiments were approved by Beth Israel Deaconess Medical Center (BIDMC) Institutional Animal Care and Use Committee (IACUC) and were performed in accordance with institutional and national guidelines.

Note that full information on the approval of the study protocol must also be provided in the manuscript.

ChIP-seq

Data deposition

☒ Confirm that both raw and final processed data have been deposited in a public database such as GEO.

☒ Confirm that you have deposited or provided access to graph files (e.g. BED files) for the called peaks.

Data access links
May remain private before publication.

https://www.ncbi.nlm.nih.gov/geo/query/acc.cgi?acc=GSE157107

Files in database submission

GSM4753165 H3K27ac-VCAP-24hr
GSM4753166 H3K27ac-VCAP-2hr
GSM4753167 H3K27ac-VCAP-Veh
GSM4753168 VCAP_H3K27AC_DHT_ENZ_REP1
GSM4753169 VCAP_H3K27AC_DHT_ENZ_REP2
GSM4753170 VCAP_H3K27AC_DHT_ENZ_REP3
GSM4753171 VCAP_H3K27AC_DHT_REP1
GSM4753172 VCAP_H3K27AC_DHT_REP2
GSM4753173 VCAP_H3K27AC_DHT_REP3
GSM4753174 VCAP_H3K27AC_FBS_REP1
GSM4753175 VCAP_H3K27AC_FBS_REP2
GSM4753176 VCAP_H3K27AC_FBS_REP3
GSM4753177 VCAP-MYC-0hr
GSM4753178 VCAP-MYC-4hr

Genome browser session
(e.g. UCSC)

http://genome.ucsc.edu/s/Haiyang/hg19_myc_paper_H.G.

Methodology

Replicates	Three replicates for "VCAP_H3K27AC_FBS", "VCAP_H3K27AC_DHT" and "H3K27AC_DHT_ENZ".
Sequencing depth	Sample Total reads Mapped reads Read type Read length<br>VCAP-MYC-0hr 62938406 59603139 SE 75bp<br>VCAP-MYC-4hr 41957242 39603897 SE 75bp<br>K27ac-VCAP-24hr 78880532 62740956 PE 150bp<br>K27ac-VCAP-2hr 96257514 74355997 PE 150bp<br>K27ac-VCAP-Veh 88197648 73332284 PE 150bp<br>VCAP_H3K27AC_DHT_ENZ_REP1 56803546 48390695 PE 150bp<br>VCAP_H3K27AC_DHT_ENZ_REP2 50977322 42177976 PE 150bp<br>VCAP_H3K27AC_DHT_ENZ_REP3 52131940 43148802 PE 150bp<br>VCAP_H3K27AC_DHT_REP1 65973476 54850959 PE 150bp<br>VCAP_H3K27AC_DHT_REP2 58819728 49706662 PE 150bp<br>VCAP_H3K27AC_DHT_REP3 114269890 89686644 PE 150bp<br>VCAP_H3K27AC_FBS_REP1 56307300 46197600 PE 150bp<br>VCAP_H3K27AC_FBS_REP2 61409966 50218758 PE 150bp<br>VCAP_H3K27AC_FBS_REP3 58258620 47730325 PE 150bp
Antibodies	MYC Santa Cruz sc-764 (ChIP-Seq, 4ug; IHC, 1:2,500)<br>H3K27Ac Abcam ab4729 (ChIP-Seq, 4ug; HiChIP, 5ug)
Peak calling parameters	bowtie2 -p 20 -x /home/group1/shared_data/gtf/Homo_sapiens_hg19/Ensembl/GRCh37/Sequence/Bowtie2index/genome -1 ${id}R1.fastq.gz -2 ${id}R2.fastq.gz 2> ./bowtie2_result/${id}.log I samtools sort -O bam-@ 20 -o ./bowtie2_result/${id}.sort.bam<br>samtools index -@ 20 ./bowtie2_result/${id}.sort.bam ./bowtie2_result/${id}.sort.bam.bai<br>macs2 callpeak -t $id --keep-dup=1 -g hs -B --SPMR -n ./macs_result/${id}_SPMR 2> ./macs_result/${id}.log
Data quality	Number of peaks with q-value < 0.05:<br>47236 Peak_K27ac-VCAP-24hr<br>35018 Peak_K27ac-VCAP-2hr<br>54375 Peak_K27ac-VCAP-Veh<br>57004 Peak_ VCAP _H3K27 AC_DHT _ENZ_REP1<br>55800 Peak_ VCAP _H3K27 AC_DHT _ENZ_REP2<br>64408 Peak_ VCAP _H3K27 AC_DHT _ENZ_REP3<br>64037 Peak_ VCAP _H3K27 AC_DHT _REP1<br>56424 Peak_ VCAP _H3K27 AC_DHT _REP2<br>74123 Peak_ VCAP _H3K27 AC_DHT _REP3<br>56822 Peak_ VCAP _H3K27 AC_FBS_REP1<br>56259 Peak_ VCAP _H3K27 AC_FBS_REP2<br>61694 Peak_ VCAP _H3K27 AC_FBS_REP3<br>15620 Peak_ VCAP-MYC-0hr<br>10011 Peak_ VCAP-MYC-4hr
Software	B0WTIE2, samtools and MACS2 were used for ChIP-Seq data analysis. The high throughput sequencing data reported in this paper have been deposited to GEO under accession number GSE157107.
